# Molecular characterisation of a bovine-like rotavirus detected from a giraffe

**DOI:** 10.1186/1746-6148-4-46

**Published:** 2008-11-13

**Authors:** Emily Mulherin, Jill Bryan, Marijke Beltman, Luke O'Grady, Eugene Pidgeon, Lucie Garon, Andrew Lloyd, John Bainbridge, Helen O'Shea, Paul Whyte, Séamus Fanning

**Affiliations:** 1 Herd and Veterinary Public Health Unit, Centres for Food Safety & Food-Borne Zoonomics, UCD Veterinary Sciences Centre, University College Dublin, Belfield, Dublin 4, Ireland; 2 National Virus Reference Laboratory, University College Dublin, Belfield Dublin 4, Ireland; 3 School of Biochemistry and Immunology, Trinity College, Dublin 2, Ireland; 4 Dublin Zoological Gardens, Phoenix Park, Dublin 3, Ireland; 5 Department of Biological Sciences, Cork Institute of Technology, Rossa Ave, Bishopstown, Cork, Ireland

## Abstract

**Background:**

Rotavirus (RV), is a member of the *Reoviridae *family and an important etiological agent of acute viral gastroenteritis in the young. Rotaviruses have a wide host range infecting a broad range of animal species, however little is known about rotavirus infection in exotic animals. In this paper we report the first characterisation of a RV strain from a giraffe calf.

**Results:**

This report describes the identification and detailed molecular characterisation of a rotavirus strain detected from a 14-day-old Giraffe (*Giraffa camelopardalis*), presenting with acute diarrhea. The RV strain detected from the giraffe was characterized molecularly as G10P[11]. Detailed sequence analysis of VP4 and VP7 revealed significant identity at the amino acid sequence level to Bovine RV (BoRV).

**Conclusion:**

This study demonstrates the need for continuous surveillance of RV strains in various animal populations, which will facilitate the identification of rotavirus hosts not previously reported. Furthermore, extending typical epidemiology studies to a broader host range will contribute to the timely identification of new emerging strain types.

## Background

Rotavirus (RV), a member of the *Reoviridae *family is one of the major etiological agents of acute viral gastroenteritis in the young of a large number of animal species including cattle, pigs, horses, rabbits, mice, dogs, cats, birds and exotic animal species such as addax, saiga, white-tailed gnu, grizzly bear, and red kangaroo [1,2]. In the developing world rotavirus infection in humans is associated with approximately 600,000 deaths per annum in those under 5 years [3]. In contrast, in the developed world RV infection is responsible for a significant economic burden being linked with 30–60% of diarrhea related hospitilisations reported [4].

RV particles are non-enveloped, icosahedral particles approximately 70 nm in diameter. The RV genome is composed of 11 double stranded RNA (dsRNA) segments, which code for 6 structural (VP1 through 4, 6 and 7) and 6 non-structural (NSP1 through NSP6) proteins. Seven classified RV groups (A-G) are recognized, based on the antigenic variability of their inner capsid protein, VP6. VP4 and VP7 (the outer capsid proteins) possess epitopes that elicit neutralizing antibody responses and in turn determine RV serotypes. Estes [5] developed a dual classification system by defining VP7-specific serotypes, termed G-types (glycoprotein) and VP4 specific serotypes termed P-types (protease sensitive protein). Genotypic classification of all RV strains is now based on this system. Based on the comparative analysis of these genes, currently there are 15 VP7 genotypes (G-types 1 through 15) and 27 VP4 genotypes (P-types [1-27]) recognized among human and animal group A rotavirus. To date, six P-types (P6[1], P7[5], P8[11], P11[14], P[17] and P[21]) and 8 G-types (G1, G3, G5, G6, G7, G8, G10 and G15) have been reported among bovine RV-group A [6-15].

Rotaviruses are intestinal pathogens that are transmitted by the faecal-oral route. In bovine animals, onset of disease is rapid and the clinical signs include depression, anorexia, diarrhea and dehydration. Large numbers of viral particles are excreted in the feces of infected animals over a period of 2 to 10 days. In temperate climates, disease is more prevalent during cooler months. In human and bovine populations, rotavirus transmission is most frequent during the winter and early spring months [16].

In previous studies it has been suggested that RV exist as mixed populations of reassortants, and this reassortment is responsible for the diversity observed between rotavirus strains. Animal RVs are often regarded as a potential reservoir for genetic exchange with human RV, due to the segmented genome structures. Co-surveillance of both animal and human RV strains is essential to gain a better understanding of the epidemiology of strains in circulation and to facilitate the timely identification of new emerging variants [17].

In this study, we report on the characterisation of a RV strain detected from a giraffe admitted to the University Veterinary Hospital with acute diarrhea. Sequence analysis revealed a significant homology to bovine RV.

## Methods

### Clinical and pathological assessment

An orphaned Rothschild giraffe from Dublin Zoological Gardens was presented to the University Veterinary Hospital at 14 days of age with a history failure of passive transfer of immunity, anorexia, dehydration, hypoglycemia, acidosis and persistent profuse watery diarrhea. The animal was treated on admission with intravenous fluid therapy, antimicrobials and non-steroidal anti-inflammatory drugs. The giraffe calf died 4 days post-treatment. A post-mortem examination primarily revealed a severe abomasitis and enteritis. No significant pathogenic organisms were isolated on bacteriological culture of the spleen, rumen or abomasum presumably as a consequence of intensive antimicrobial therapy. A faecal specimen was sent to our laboratory for further investigation.

Preliminary testing of the faecal specimen obtained (by Transmission Electron Microscopy) revealed the specimen to be positive for RV infection. Husbandry issues were considered to assess the potential route of RV infection. Interestingly, the calf had not been in contact with any other ruminants' prior to admission to the University Veterinary Hospital. However indirect contact with other animals could not be out ruled. Other issues for consideration were the movements of the keepers between different animal enclosures, the feeding equipment used and the apparatus used to clean the housing areas.

### Transmission electron microscopy (T-EM)

Transmission electron micrographs captured the appearance of negatively stained RV observed in the giraffe faecal sample. Faecal samples were prepared by mixing with 2% (w/v) methylamine tungstate negative stain on a 3.05 mm grid and allowed to stand at room temperature for 4 min. The specimen was examined using electron-optical magnification up to × 40, 000 range.

### Extraction of viral dsRNA

A faecal specimen from the 14-day old giraffe was sent to our laboratory for analysis. Initially this sample was diluted in phosphate buffered saline solution (1× PBS). Rotavirus dsRNA was purified from the faecal specimen using a standard phenol/chloroform protocol with ethanol precipitation [18]. Briefly, 320 μl of the faecal suspension above was combined with 40 μl 10% (w/v) SDS and 40 μl 10% (w/v) proteinase K and incubated for 90 min at 37°C. The suspension was extracted once with phenol/chloroform (5:1) followed by one extraction in chloroform alone. RNA in the aqueous phase was removed to a clean eppendorf tube and precipitated with two volumes of ethanol and then stored at -20°C overnight. Purified dsRNA was recovered by centrifugation (1, 000 × g for 30 min) and the pellet containing the viral nucleic acid was suspended in 100 μl diethyl pyrocarbonate (DEPC) treated water. Purified dsRNA was stored at -80°C until required.

### SDS-PAGE

dsRNA segments were separated by electrophoresis in 8% (w/v) polyacrylamide slab gels, 1.5 mm thick with a 7.3 cm path length. Electrophoresis was performed using a discontinuous buffer system based on a modification previously described by Laemmli for 80 min at a constant voltage of 110 V [19].

The presence of viral dsRNA in the faecal sample was confirmed following staining with a DNA Silver Staining Kit according to the manufacturer's instructions (Amersham Biosciences, UK).

### Reverse Transcriptase-Polymerase Chain Reaction

A one-step reverse transcriptase-polymerase chain reaction (RT-PCR) was performed. Purified viral genomic dsRNA (3 μl, containing approx. 50 ng) was added to 3.5 μl DMSO (Sigma-Aldrich, Steinheim, Germany), and denatured by heating for 5 min at 94°C. The tubes were placed immediately on ice to prevent re-annealing of dsRNA. RT-PCR was performed according to the modified methods described by Gentsch *et al.*, [20] and Gouvea *et al.*, [21] (Table 1) using 30 thermal cycles at 94°C for 1 min, 55°C; for 2 min, 72°C for 2 min followed by 10 min incubation at 72°C.

**Table 1:** Primers used for VP4, VP7 amplification and subsequent P and G typing

**Target**	**Sequence (5'→ 3')**	**Position**	**Size (bp)**	**Reference**
VP4 gene-con2	ATT TCG GAC CAT TTA TAA CC	11–32		[20]
VP4 gene-con3	TGG CTT CGC TCA TTT ATA GAC A	887–868	877	[20]
P1	CGA ACG CGG GGG TGG TAC TTG	269–289	619	[20]
P5	GCC AGG TGT CGC ATC AGA G	336–354	555	[20]
P11	GGA ACG TAT TCT AAT CGC GTG	574–594	314	[20]
VP7-End9 (UK)	GGT CAC ATC ATA CAA CTC TAA TCT	1062–1036		[22]
VP7-Beg9	GGC TTT AAA AGA GAG AAT TTC CGT TTG	1–28	1062	[21]
G6	CTA GTT CCT GTG TAG AAT C	499–481	499	[23]
G8	CGG TTC CGG ATT AGA CAC	273–256	273	[23]
G10	TTC AGC CGT TGC GAC TTC	714–697	714	[23]
G5	CAT GTA CTC GTT GTT ACG TC	779–760	779	[23]
G11	GTC ATC AGC AAT CTG AGT TGC	336–316	336	[23]

Briefly, denatured dsRNA was added to a reaction mixture consisting of 10 μl 5 × reaction buffer (Promega, Madison, WI, USA), 8 μl of a deoxyonucleoside triphosphate mixture (consisting of 1.25 mM of each dNTP) (Promega), 3 μl 25 mM MgCl_2 _(Promega), 0.1 μl reverse transcriptase from avian myeloblastosis virus (5 U/μl) (Promega), 0.5 μl *Taq *DNA polymerase (5 U/μl) (Promega,) and 1 μl of each selected primer pair (20 pM) as detailed in Table 1. VP4 and VP7 amplification reactions produced DNA amplicons of ~880 and 1,062 bp respectively.

### Multiplex semi-nested PCR

A semi-nested, multiplex PCR reaction was performed using primers targeting the genotype-specific regions of the VP4 and VP7 genes [20-24].

#### G-type determination

Two μl (of a 1:100 dilution of the first round PCR product) was used as the template from the second round of amplification along with 10 μl of 5 × reaction buffer, 6 μl 25 mM MgCl_2_, 2 μl dNTP (1.25 mM final concentration), 0.5 μl DNA *Taq *polymerase (5 U/μl), 1 μl of the 5'-common forward primer (20 pM) and 1 μl each of primers specific for bovine G-types (G5, G6, G8, G10 and G11) as reported previously by Gouvea *et al*., [22,23]. Amplification was performed using modified conditions described by Falcone *et al*., [24]. Briefly, the thermal profile consisted of a 2 min incubation step at 94°C, followed by 30 PCR cycles at 94°C for 30 sec, 42°C for 30 sec and 72°C for 45 sec and a final extension period for 7 min at 72°C.

#### P-type determination

Second round amplifications were carried out using Con2 and Con3 consensus primers and three different sub-typing primers (Table 1), as previously described by Gouvea *et al.*, [21].

First and second round amplicons were resolved in a 1.5% (w/v) molecular biology grade agarose gel, stained with 0.1 mg/ml ethidium bromide and viewed under ultraviolet light.

### DNA Sequencing and phylogenetics analysis

Amplified DNA fragments were purified using a QIAGEN PCR purification Kit (QIAGEN, Hilden, Germany) and sequenced in both directions. Nucleotide sequences were assembled and analysed by DNAStar software (Lasergene, Madison, WI). All sequences were compared against those available in the current GenBank database . Sequence data from the giraffe samples were entered in the GenBank database under the following Accession numbers [GenBank: EU548032 and GenBank: EU548033].

Separate alignments for the Giraffe VP7 and partial length VP4 amino acid sequences were performed. The alignments were imported into Mega4 and neighbour-joining trees were calculated with Poisson correction, deleting all sites where a gap appeared in any sequence. Statistical confidence was established by carrying out 1000 bootstrap replicates and branches were collapsed unless supported by 80% of the bootstraps [25-28].

## Results

### Preliminary characterisation of the giraffe faecal specimen

**Figure 1:**
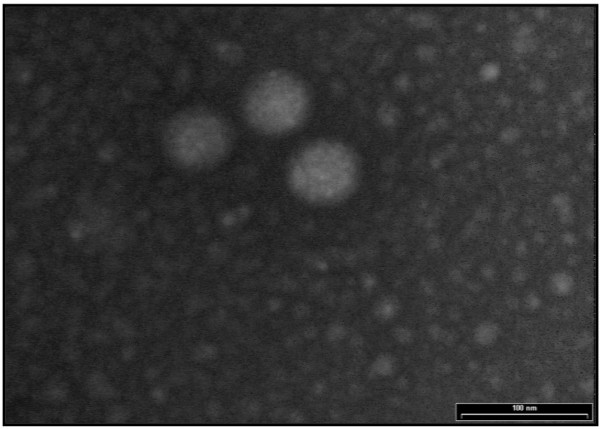
**A transmission electron micrograph**. The image shows the appearance of negatively stained RV detected from the giraffe faecal sample. RV particles are approximately 70 nm in diameter. (A 100 nm size bar is included for comparison).

The presence of RV in the faecal specimen obtained from the 14-day old giraffe was assessed initially by T-EM. Clusters of negatively stained RV particles with an average diameter of 70 nm were observed (Fig. 1) in the electron micrographs. These particles were similar in shape when compared to those RV of human and bovine origin (data not shown).

**Figure 2:**
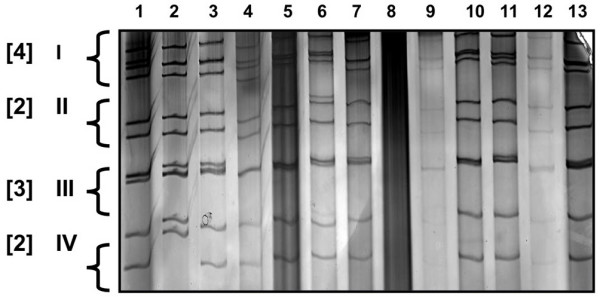
**SDS-PAGE demonstrating electrophoresis pattern of GirRV**. dsRNA segments were separated by electrophoresis in 8% (w/v) polyacrylamide slab gels. Lane 1: Human RV-1 (HRV), Lane 2: HRV-2, Lane 3: HRV-3, Lane 4: HRV-4, Lane 5: HRV-5, Lane 6: HRV-6, Lane 7: GirRV, Lane 8: -ve control, Lane 9: Bovine RV-1, Lane 10: Bovine RV-2, Lane 11: Bovine RV-3, Lane 12: Bovine RV-4, Lane 13: Bovine RV-5.

RV in the faecal specimen was confirmed after purification and subsequent analysis of the dsRNA banding profile in a SDS-PAGE gel. The electropherotype obtained was compared against several unrelated RV strains obtained from other animal and human sources (Fig. 2). The profile for Giraffe rotavirus (GirRV) (Fig. 2, lane 7), displayed the typical group A rotavirus dsRNA genome banding pattern (consisting of a 4, 2, 3, 2 arrangement) characteristic of the 'long' RNA electropherotype pattern.

**Figure 3:**
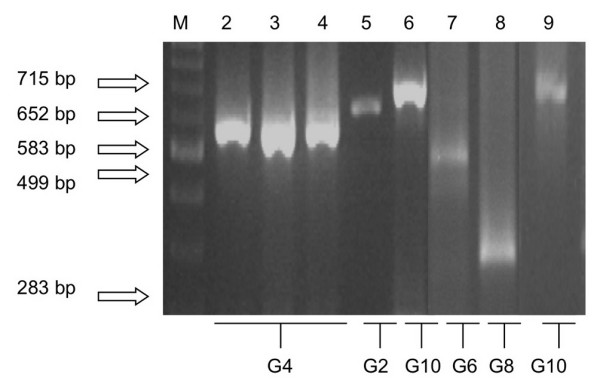
**G-type determination showing a selection of human and bovine RV samples**. (Amplicon sizes are shown in parenthesis). A 1.5% (w/v) agarose gel in a 1 × TBE buffer stained with 0.1 mg/ml of ethidium bromide. Lane 1: 100 bp Marker (New England Biolabs), Lane 2 through 4: G4 (HRV-1 – 583 bp), Lane 5: G2 (HRV-2 – 652 bp), Lane 6: G10 (Bovine RV-3–715 bp), Lane 7: G6 (Bovine RV-1 – 499 bp), Lane 8: G8 (Bovine RV-2 – 283 bp), Lane 9: G10 (GirRV – 715 bp)

The identification of the corresponding G- and P-types were established by RT-PCR on the purified dsRNA from the giraffe sample (using primers listed in Table 1). The complete VP7 gene was amplified using a pair of broadly specific consensus primers, located at the beginning (1–21 nt) and end (1,062–1,036 nt) of this gene. In a semi-nested PCR, using a set of internal specific primers, the G-type was determined as G10 (Fig. 3, lane 9).

**Figure 4:**
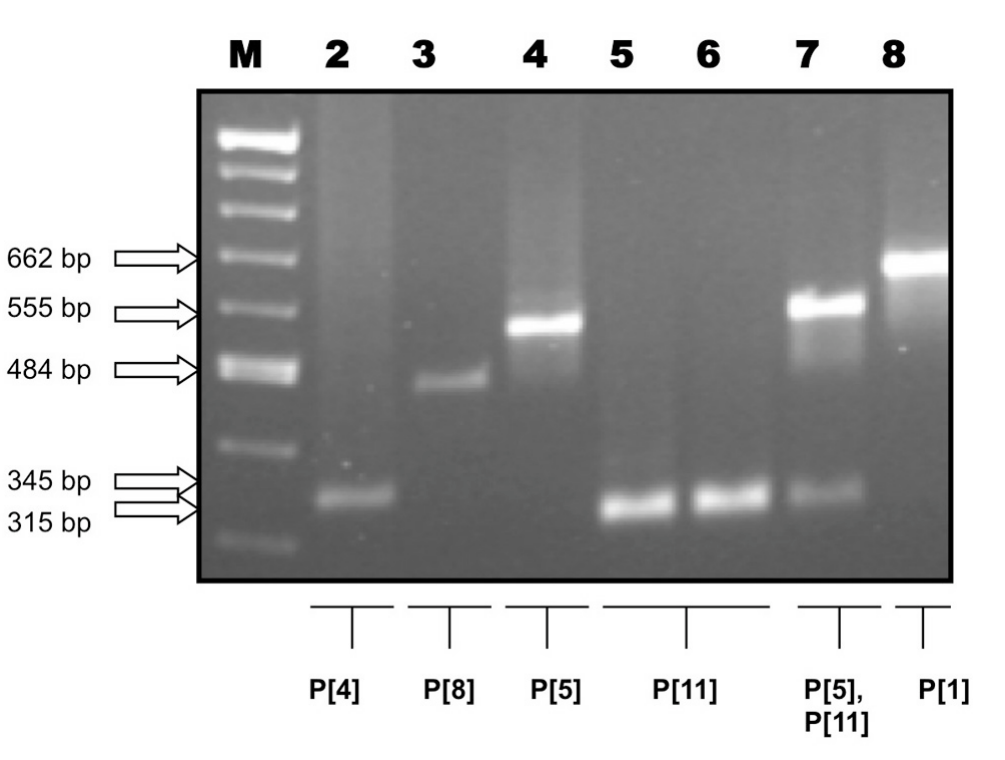
**P-type determination showing a selection of human and bovine RV samples**. (Amplicon sizes are shown in parenthesis). A 1.5% agarose gel was used to visualize P-typing amplicons from a semi-nested multiplex PCR reaction. Lane 1: 100 bp Marker (New England Biolabs), Lane 2: P[4] (HRV-1 – 345 bp), Lane 3: P[8] (HRV-2 – 484 bp), Lane 4: P[5] (Bovine RV-1 – 555 bp), Lane 5: P[11] (Bovine RV-2 – 315 bp), Lane 6: P[11] (GirRV-315 bp), Lane 7: P [5+11] (Bovine RV-3 – 555, 315 bp), Lane 8: P[1] (Bovine RV-4 – 662 bp).

Similarly, partial gene 4 sequence was amplified and subsequently genotyped using primers for the detection of widely distributed bovine P-types. These data identified a P[11] genotype (Fig. 4, lane 6).

The genotypes for giraffe rotavirus (GirRV) detected in the faecal specimen were classified as G10P[11].

### Sequence and phylogenetic analysis

Phylogenetic analysis of the amino acid sequences derived from VP4 and VP7 genes was undertaken, using a set of similar genes selected from the current databases, for comparison purposes. Comparisons with the VP4 and VP7 sequence from the giraffe sample revealed closest similarities to bovine RV sequences. Alignment studies (data not shown) showed a 98% identity at the amino acid sequence level between giraffe and bovine species.

**Figure 5:**
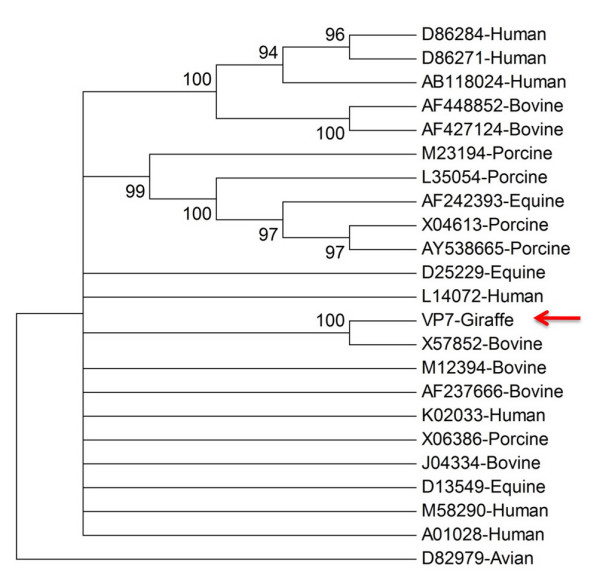
**Evolutionary relationships of 23 VP7 proteins**. The evolutionary history was inferred using the Neighbor-Joining method [25], implemented in MEGA4 [26]. The percentage of replicate trees in which the associated taxa clustered together in the bootstrap test (1000 replicates) are shown next to the branches [27]. Branches with less than 80% bootstrap support have been collapsed. The evolutionary distances were computed using the Poisson correction method [28] and are in the units of the number of amino acid substitutions per site. All positions containing gaps and missing data were eliminated from the dataset (Complete deletion option). There were a total of 293 positions in the final dataset. The Giraffe sequence is identified with the arrow as shown in the figure.

The full length VP7 amino acid sequence was analysed and compared to 15 well-established G-genotypes previously published. GirRV was most similar to a bovine G10 gene [Accession number GenBank: X57852] (Fig. 5 and Table 2).

**Table 2:** The origins of strains and their respective VP7 G-genotypes used to construct Figure 5

**RV Strain (Origin)**	**GenBank Accession number**	**G-type**
Wa (Human)	K02033	G1
Hu/5 (Human)	A01028	G2
Yo (Human)	D86284	G3
AU-1 (Human)	D86271	G3
P (Human)	AB118024	G3
CP-1 (Bovine)	AF448852	G3
PP-1 (Bovine)	AF427124	G3
Gottfried (Porcine)	X06386	G4
OSU (Porcine)	X04613	G5
JL49 (Porcine)	AY538665	G5
H-1 (Equine)	AF242393	G5
A46 (Porcine)	L35054	G5
NCDV (Bovine)	M12394	G6
PO-13 (Avian)	D82979	G7
B37 (Bovine)	J04334	G8
116E (Human)	L14072	G9
B223 (Bovine)	X57852	G10
YM (Porcine)	M23194	G11
L26 (Human)	M58290	G12
L338 (Equine)	D13549	G13
CH3 (Equine)	D25229	G14
Hg18 (Bovine)	AF237666	G15

**Figure 6:**
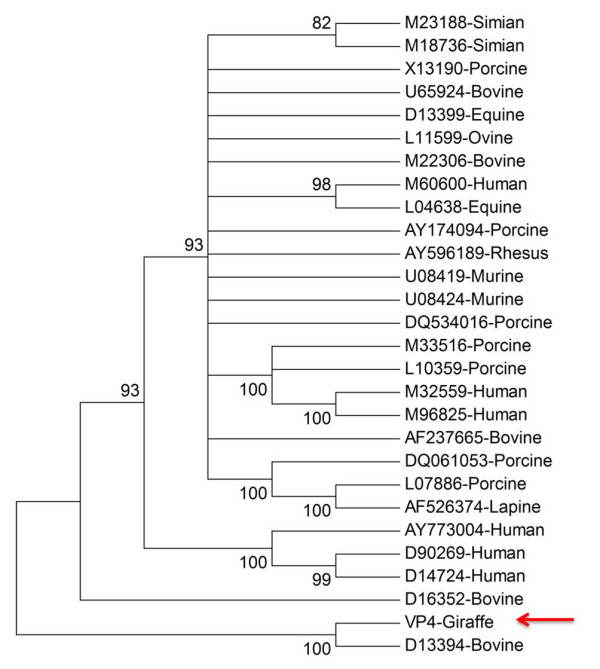
**Evolutionary relationships of 28 VP4 proteins**. The evolutionary history was inferred using the Neighbor-Joining method [25], implemented in MEGA4 [26]. The percentage of replicate trees in which the associated taxa clustered together in the bootstrap test (1000 replicates) are shown next to the branches [27]. Branches with less than 80% bootstrap support have been collapsed. The evolutionary distances were computed using the Poisson correction method [28] and are in the units of the number of amino acid substitutions per site. All positions containing gaps and missing data were eliminated from the dataset (Complete deletion option). There were a total of 229 positions in the final dataset. The Giraffe sequence is identified with the arrow as shown in the figure.

Comparison of the partial VP4 amino acid sequence of GirRV with 27 P-genotypes previously reported for group A rotaviruses revealed the closest branching relationship to the bovine P[11] type, [Accession number GenBank: D13394] (Fig. 6 and Table 3).

**Table 3:** The origins of strains and their respective VP4 P-genotypes used to construct Figure 6

**RV Strain (Origin)**	**GenBank Accession number**	**P-type**
RF (Bovine)	U65924	P[1]
SA11 (Simian)	M23188	P[2]
RRV (Simian)	M18736	P[3]
RV-5 (Human)	M32559	P[4]
UK (Bovine)	M22306	P[5]
Gottfried (Porcine)	M33516	P[6]
OSU (Porcine)	X13190	P[7]
Wa (Human)	M96825	P[8]
K8 (Human)	D90269	P[9]
69M (Human)	M60600	P[10]
B223 (Bovine)	D13394	P[11]
H-2 (Equine)	L04638	P[12]
MDR-13 (Porcine)	L07886	P[13]
PA169 (Human)	D14724	P[14]
Lp14 (Ovine)	L11599	P[15]
EB (Murine)	U08419	P[16]
993–83 (Bovine)	D16352	P[17]
L338 (Equine)	D13399	P[18]
4F (Porcine)	L10359	P[19]
EHP (Murine)	U08424	P[20]
Hg18 (Bovine)	AF237665	P[21]
160/01 (Lapine)	AF526374	P[22]
A34 (Porcine)	AY174094	P[23]
TUCH (rhesus macaque)	AY596189	P[24]
Dhaka6 (Human)	AY773004	P[25]
134/04 – 15 (Porcine)	DQ061053	P[26]
CMP034 (Porcine)	DQ534016	P[27]

## Discussion

In this report we describe the identification and subsequent molecular characterisation of a RV strain detected from a 14-day old Rothschild giraffe with acute diarrhea. To our knowledge, this is the first report of the detection of RV in a giraffe. Phylogenetic analysis showed that the GirRV strain was closely related to bovine RV strains. There is limited information on the prevalence of RV in zoo environments and exotic species. In 1978, Eugster and colleagues described the clinical and laboratory findings of an outbreak of pneumoenteric disease in a zoo nursery [29]. This was the first study of its kind to report a RV-associated infection in zoo animals. More recent studies identified RV in a variety of exotic species in their natural habitats and zoo nurseries [30-33]. Petric and colleagues [30] identified a wide range of animals that were sero-positive for RV in zoo environments. In Peru, Rivera *et al.*, [31] examined sera from alpacas (*Lama pacos*) for antibodies to 8 viruses known to infect other domestic animals. On the basis of these data and additional supporting clinical information Rivera *et al. *concluded that RV infects alpacas. Puntel *et al. *[32] reported a serological survey that investigated for a variety of viruses in llamas on Argentinean farms. Samples taken in this study were tested for antibodies against viruses known to infect cattle (including bovine rotavirus). Results showed that 87.69% of llamas tested positive for bovine RV antibodies. More recently Parreno *et al.*, [34] reported the G- and P-types of two RV strains isolated from newborn guanacos (*Lama guanicoe*) that presented with acute diarrhoea in Argentina. Group A RV with a G8 genotype was identified. Phylogenetic analysis of these RV strains, showed a close relationship to other G8 bovine RV previously reported in Japan, the USA and Switzerland. The P-types identified in this study, included the common P[1] and an unusual P[14] type, related to human and goat P[14] strains. This was the first report of a G8P[14] strain in Argentina [34].

In our study, we employed RT-PCR based genotyping methods to successfully identify the G- and P-type associated with the RV strain from the giraffe fecal specimen. SDS-PAGE analysis showed that the GirRV possessed a 'long' electropherotype pattern, typical of the majority of RV strains from human and animal sources. The VP7 and VP4 genes were sequenced and compared to a selection of the corresponding genes of human and bovine origin. Sequence comparisons showed a close genetic similarity to RV strains reported previously in bovine animals.

Epidemiological studies of rotavirus infections are increasingly revealing a diversity of strains co-circulating in the human and animal populations worldwide. This strain diversity may be due to two mechanisms – the accumulation of point mutations (genetic drift), which generates genetic lineages and leads to the emergence of antibody escape mutants, and genetic shift, operating through gene reassortment arising from dual infection of a single cell [34].

Rotavirus G10P[11] strains, are commonly found in cattle and have frequently been associated with asymptomatic neonatal infections in India [35]. Indeed G10P[11] has also been detected in a minority of bovines in Ireland [36]. Therefore, studying the distribution of rotavirus G- and P-types among various animal species is important to improve our understanding of RV epidemiology and the mechanisms by which these viruses evolve, cross the species barriers, exchange genes during reassortment, and mutate *via *the accumulation of single-nucleotide polymorphisms and/or other genetic rearrangements.

## Conclusion

This is the first study to report the molecular characterization of rotavirus in a giraffe calf. These data underpins the necessity for continuous surveillance of rotavirus in animal and human populations to improve and extend our understanding of potential zoonotic links.

## Authors' contributions

EM was responsible for carrying out this study and was the main contributor to writing the manuscript. LG and AL carried out the bioinformatic analysis on sequences. EP provided electron micrograph images of rotaviral particles detected in faecal specimen. MB, JB, and LO'G and JB provided veterinary expertise for the study. SF, PW and HO'S co-ordinated the project and reviewed all drafts of the manuscript. All authors read, commented on and approved the final manuscript.
